# Extracellular vesicles in low volume uterine lavage and serum: novel and promising biomarker for endometritis in Arabian mares

**DOI:** 10.1186/s12917-022-03137-3

**Published:** 2022-01-18

**Authors:** Sally Ibrahim, Mohamed Hedia, Mohamed O. Taqi, Mohamed K. Derbala, Karima Gh. M. Mahmoud, Youssef Ahmed, A. S. Sosa, Yasser H. A. Saber, M. H. Hasanain, M. F. Nawito, George E. Seidel

**Affiliations:** 1 Department of Animal Reproduction and A.I, Veterinary Research Division, National Research Centre, Dokki, Giza, 12622 Egypt; 2 Department of Theriogenology, Faculty of Veterinary Medicine, Cairo University, Giza, Egypt; 3 Central Laboratory for Agricultural Climate, Agricultural Research Centre, Ministry of Agriculture and Land Reclamation, Dokki, Giza, 12311 Egypt; 4 Animal Reproduction Research Institute, Diagnostic Imaging and Endoscopy Unit, Giza, Egypt; 5 Animal Reproduction and Biotechnology Laboratory, Colorado State University, Fort Collins, CO USA

**Keywords:** Extracellular vesicles, Mares, Endometritis, Uterine lavage, Serum, Interleukin 6, Prostaglandins

## Abstract

**Background:**

Extracellular vesicles (EVs) are a promising biomarker and play a vital role in cell–cell communication. This study aimed (I) to identify and characterize EVs from low volume uterine lavage (LVL) and serum in mares with endometritis, compared to healthy controls and (II) to measure serum levels of interleukin 6 (IL-6), and prostaglandins (PGF_2α_ and PGE_2_). Mares were divided into 30 sub-fertile (endometritis) and 20 fertile (controls). Serum and LVL was collected for EV isolation, and determination of serum levels of inflammatory mediators. Characterization and visualization of EVs were done by electron microscopy, dynamic light scattering and flow cytometry.

**Results:**

Serial ultracentrifugation of LVL and use of a commercial kit for serum were strategies for EVs isolation. Mares with endometritis released higher amounts of larger size EVs. The EVs from mares with endometritis differentially expressed CD9 and CD63, compared to controls. Mares suffering from endometritis evoked higher levels of inflammatory mediators.

**Conclusions:**

Thus, EVs could be used for a better understanding the regulatory mechanisms associated with developing endometritis in mares.

**Supplementary Information:**

The online version contains supplementary material available at 10.1186/s12917-022-03137-3.

## Background

In the recent years, an increasing number of studies indicated a role of extracellular vesicles (EVs) in animal reproduction and fertility [1, 2]. It was mentioned that EVs are evolutionarily conserved (from bacteria to plant, animal and humans), and they are derived from cells via the endosomal system or shedding from plasma membrane [3, 4]. The EVs are three categories; microvesicles, exosomes, and apoptotic bodies according to their cellular origin [3, 5]. Microvesicles (MVs) form directly from the outward budding of the plasma membrane, and their size (~ 100–1000 nm) [6, 7]. Exosomes are smaller in their size (~ 50–120 nm), and they undergo a complex process that encloses inward budding of endosomes [6, 8]. It was reported that dying cells release apoptotic bodies (500 nm–2 μm), which might be more abundant than MVs or exosomes, under certain circumstances, so they are variable in size, structure, as well as composition [9]. They are found in biological fluids such as plasma, semen, amniotic fluid, milk, saliva, uterine luminal fluid, and urine [1, 2]. Moreover, EVs play a vital role in many physiological and pathological processes [5]. It is well documented that EVs are secreted by different reproductive tissues such as; ovarian, oviductal, and uterine tissues, which reflects their significant role in cell-cell communications, during follicular development, fertilization, and pregnancy (conceptus-endometrial interface) [1, 10]. The range of their diameters is 50 to 1000 nm [3]. It was reported that tetrespanins (CD9, CD63, and CD81), other proteins {ALG-2 interacting protein X (ALIX) and tumour susceptibility gene 101 protein (TSG101)} are specific surface marker as well as the hallmark of EVs [3, 11]. Interestingly, EVs have certain receptors and/or ligands from the original cells, and this reveals their potential for selective interactions with specific target cells [12, 13]. Furthermore, these vesicles are mainly responsible for intercellular communication, through exchange biological materials such as nucleic acids (DNA, mRNA, and miRNA), lipids and proteins [3, 5]. The EVs can horizontally transfer those genetic materials to other cells, which can then alter the functions of the recipient cell [14]. They play a vital role in modulating the immune system and thus for inflammatory immune response [15]. Therefore, the accumulating evidence indicates that EVs are a promising strategy for investigation of therapeutic targets, understanding interactions between host and pathogen, as well as selection of biomarkers (sensitive, accurate and specific) for different diseases [16].

Equine endometritis, a local inflammation of the superficial layers of the uterus, has been ranked as the third most common medical problem of adult horses by equine practitioners [17]. The decrease in pregnancy rates in affected mares causes significant losses to the horse breeding industry [18, 19]. Uterine infection does not only affect female fertility by perturbing uterine function, but also could prolong ovarian cycle [20]. Endometritis is a major cause of mare infertility that arise from failure to remove bacteria, spermatozoa and inflammatory exudate post-breeding, and it is often underdiagnosed if occurring in a subclinical form i.e. without clinical signs [21]. Reproductively sound mares respond to these contaminants with a definite immune response through activation of various humoral (pro-inflammatory cytokines) and cellular (phagocytic cells) agents [22, 23]. However, mares that fail to properly evoke such systemic inflammatory reaction are more likely to be susceptible for endometritis [21, 24]. Endometritis provides the uterine environment with unsuitable conditions for sperm cells and implantation of the embryo [25]. In the last decades, there were several attempts to properly trace and control the pathophysiological pattern of equine endometritis. In addition, researchers conducted very promising prognostic and diagnostic techniques for endometritis in mares, which precisely help in the management of such crucial conditions. These techniques were mainly explored through checking various cytological, microbiological, ultrasonographical and serological biomarkers in mares suffering from endometritis [25–27]. However, these different methodologies did not achieve better understanding of molecular regulation of equine endometritis. It is well documented that cytokines and prostaglandins are key players in different reproductive processes and their dysregulation might perturb normal uterine functions [26, 28–30]. Many studies showed that endometritis in mares was associated with clear changes in the expression pattern of pro-inflammatory cytokines such as IL-6 and prostaglandins in the uterine tissue samples [23, 28, 31]. Recently, it was reported that serum concentrations of IL-6 and prostaglandins (PGF_2α_ and PGE_2_) were higher in mares suffering from endometritis compared to healthy ones [29, 32]. Recently, it was reported that EVs could be exploited by cytokines/chemokines (non-canonical pathway) to be released into the extracellular milieu. Thus, they could subsequently affect physiological and/or pathological function of target cells [33].

Previously, the role of EVs in follicular fluid and serum during follicular development and pregnancy, was studied in mares [34, 35]. In a very recent study, Alminana et al. [36] and her co-workers successfully isolated EVs from uterine fluid of mares with a good yield and purity, which could be used for future research work, in order to study their role during embryo-maternal interactions. To the best of our knowledge, the role of EVs in uterine lavage and serum during endometritis in mares was not yet investigated. Therefore, we hypothesized that the identification and characterization of EVs and their cargo could be exploited for a better understanding of the molecular regulation of equine endometritis and subsequently choose the best diagnostic as well as intervention tools. Thus, the current work aimed to identify and characterize EVs from low volume uterine lavage (LVL) and serum in mares with endometritis, in comparison with normal healthy (control) ones. Moreover, the concentrations of interleukin 6 (IL-6), and prostaglandins (PGF_2α_ and PGE_2_) were measured in serum. This study could be a forward step for proper understanding of the interactions between EV content (cargo) and inflammatory mediators (local as well as systemic), to maintain the tight balance between multiple pro- and anti-inflammatory mediators, which are required for rapid and efficient elimination of bacteria and subsequently enhance normal uterine function (receptivity & cyclicity) in Arabian mares.

## Results

### The morphology and size distribution of EVs

**Fig. 1:**
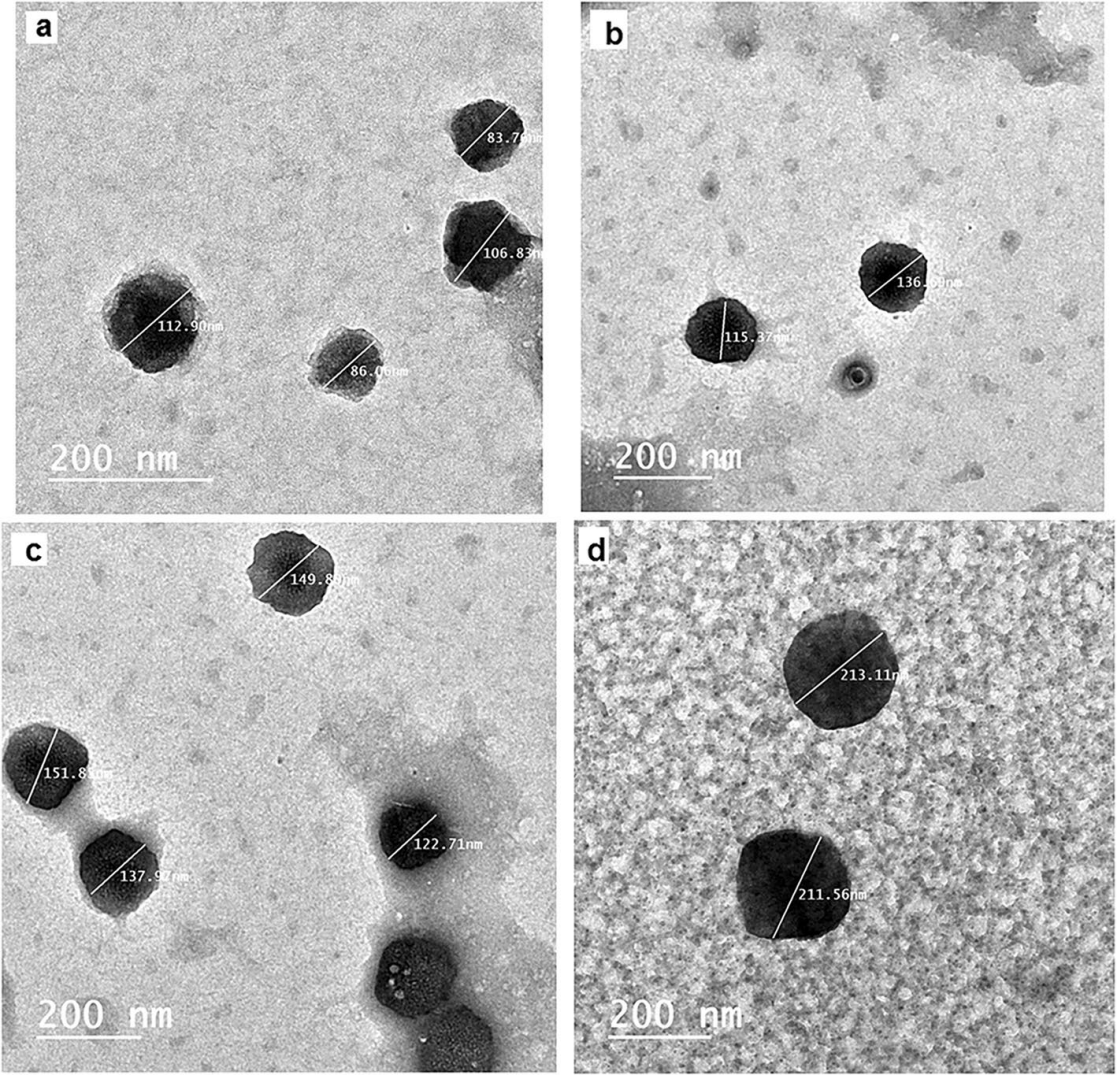
Transmission electron microscopic (TEM) visualization of EVs isolated from serum and LVL of healthy (control) and diseased (endometritis) Arabian mares. **A** Isolated EVs from serum by a commercial kit of the control group. **B** Isolated EVs from LVL by serial ultracentrifugation of the control group. **C** Isolated EVs from serum by a commercial kit of the diseased (endometritis) group. **D** Isolated EVs from LVL by serial ultracentrifugation of the diseased (endometritis) group

**Fig. 2:**
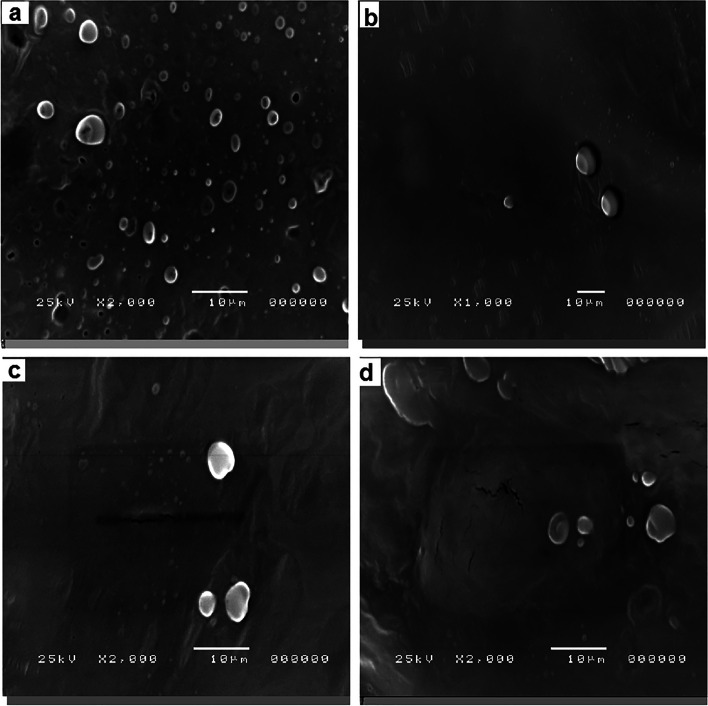
Scanning electron microscopic (SEM) visualization of EVs isolated from serum and LVL of healthy (control) and diseased (endometritis) Arabian mares. **A** Isolated EVs from serum by a commercial kit of the control group. **B** Isolated EVs from LVL by serial ultracentrifugation of the control group. **C** Isolated EVs from serum by a commercial kit of the diseased (endometritis) group. **D** Isolated EVs from LVL by serial ultracentrifugation of the diseased (endometritis) group

The images obtained by electron microscopy confirmed the presence of EVs in serum and LVL of control and diseased groups. The morphology and size of EVs in LVL and serum were confirmed by electron microscopy; TEM (Fig. 1A-D) and SEM (Fig. 2A-D). The TEM images revealed cup-shaped morphology, which is the most characteristic feature of EVs. Furthermore, the images acquired by TEM and SEM showed that EVs (from serum and LVL) are heterogeneous in shape, in both groups; control (Fig. 1A&B), (Fig. 2A&B) and diseased (Fig. 1C&D) and (Fig. 2C&D). The larger vesicles were most frequent in the diseased group (endometritis), for both serum (122.71–151.8 nm), (Fig. 2C) and LVL (211.56–213.11 nm), (Fig. 2D). While, the smaller ones were noticed in control group: serum (83.7–112.9 nm), (Fig. 1A) and LVL (115. 37–136.69 nm), (Fig. 1B).

**Fig. 3:**
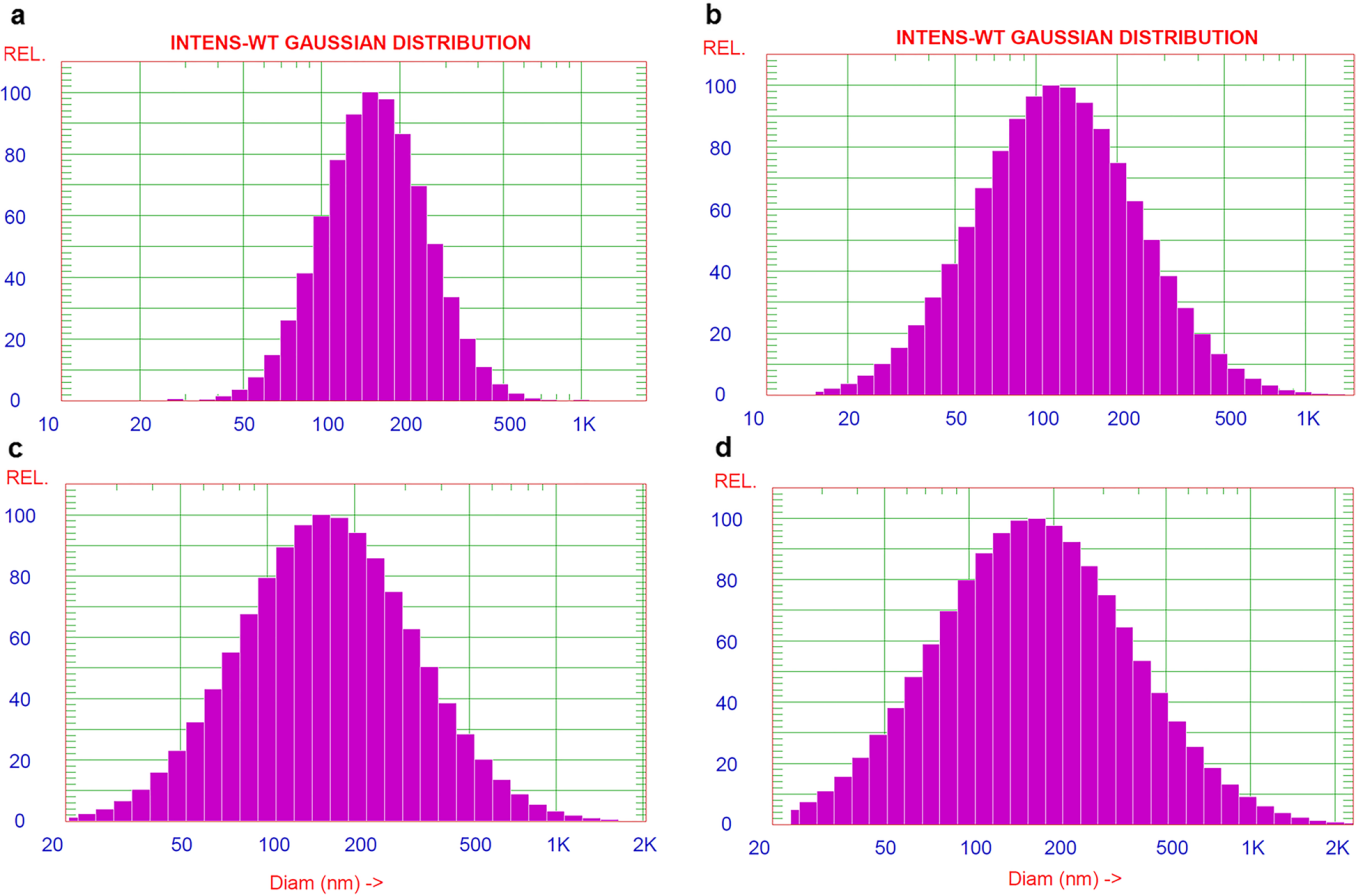
Dynamic Light Scattering measurements and particle size distribution of EVs isolated from serum and LVL of healthy (control) and diseased (endometritis) Arabian mares. **A** Size distribution of isolated EVs from serum by a commercial kit of the control group. **B** Size distribution of isolated EVs from LVL by serial ultracentrifugation of the control group. **C** Size distribution of isolated EVs from serum by commercial kit of the diseased (endometritis) group. **D** Size distribution of isolated EVs from LVL by serial ultracentrifugation of the diseased (endometritis) group

**Fig. 4:**
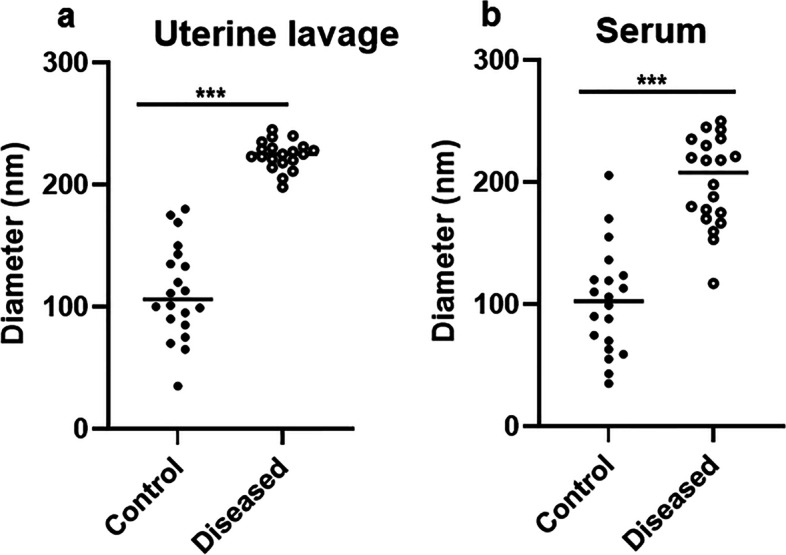
EV size distribution in serum and LVL of healthy (control) and diseased (endometritis) Arabian mares. **A** Size distribution of isolated EVs from LVL in mares suffering from endometritis compared to healthy controls. **B** Size distribution of isolated EVs from serum in mares suffering from endometritis compared to healthy ones. Data presented as mean ± SEM (five replicates/isolation methods). ^***^: Statistically significant at *P* < 0.001

The results of DLS showed that EVs in serum and LVL exhibited a clear asymmetric size distribution between control and diseased mares (Fig. 3A-D). It was also noticed that the size of EVs in both LVL and serum was larger (*P* < 0.001), (224.2 ± 2.58 nm and 200 ± 8.22 nm, respectively) in mares with endometritis compared to control healthy ones (112.2 ± 8.65 nm and 101.8 ± 9.77 nm, respectively), (Fig. 4A&B).

### Marker-based assessment of isolated EVs

The typical surface markers (CD63 and CD81) of EVs were clearly detected by the enzyme-linked immunosorbent assay (ELISA) kits. The standard curve indicated that the isolated EVs from serum and LVL in both control and diseased mares expressed a high level of CD63 and CD81 (Supplementary Figs. 1 and 2).

**Fig. 5:**
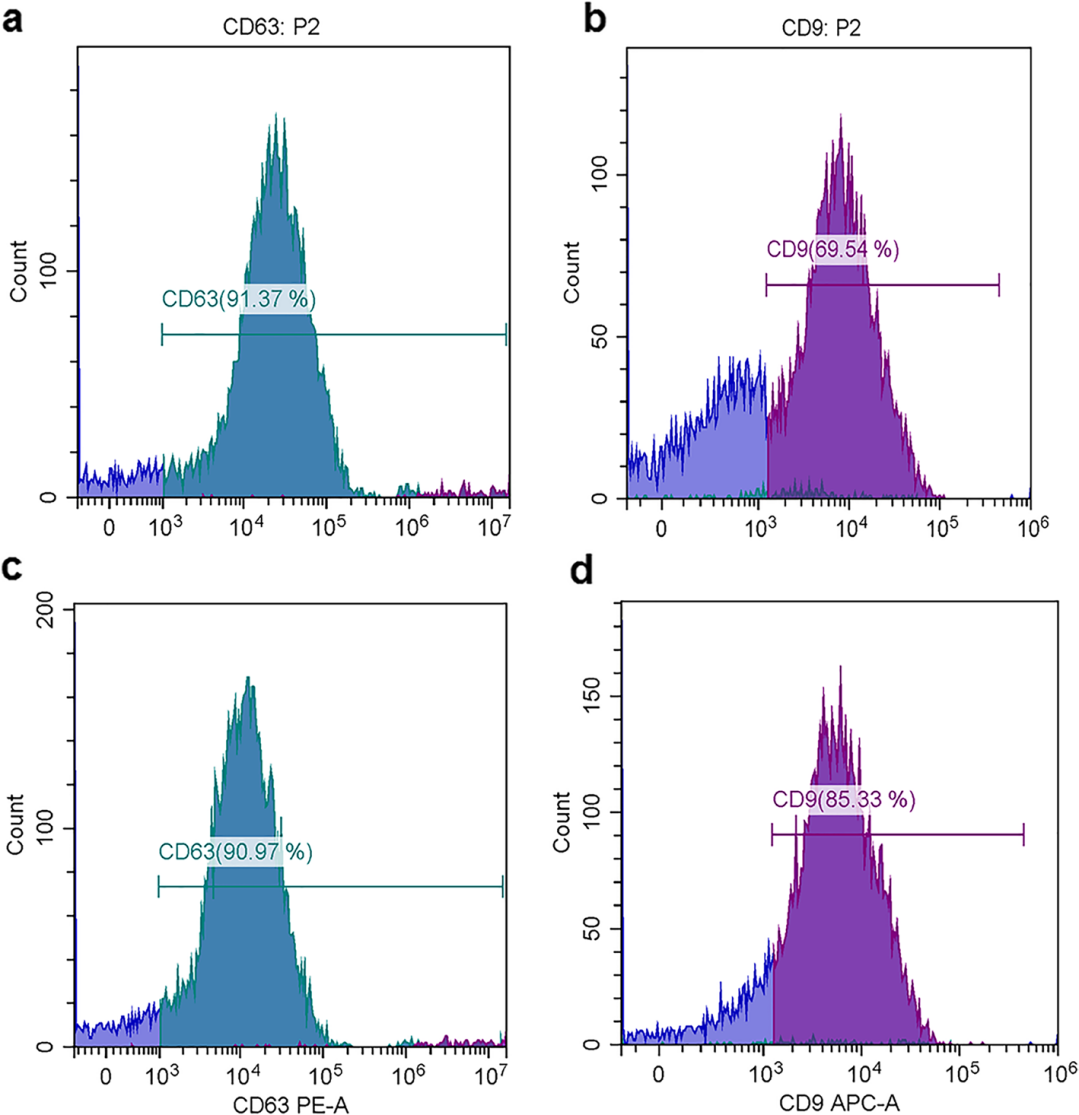
Flow cytometric analysis of tetraspanins (CD63 and CD9) expression in EVs subtypes isolated from serum of healthy (control) and diseased (endometritis) Arabian mares. **A** Histogram representative of CD63 expression in EVs subtypes isolated from serum of healthy (control) mares. **B** Histogram representative of CD9 expression in EVs subtypes isolated from serum of healthy (control) mares. **C** Histogram representative of CD63 expression in EVs subtypes isolated from serum of diseased (endometritis) mares. **D** Histogram representative of CD9 expression in EVs subtypes isolated from serum of diseased (endometritis) mares. Fluorescence (CD63-PE-A and CD9-APC-A, x-axis), vs. number of events (Count, y-axis)

**Fig. 6:**
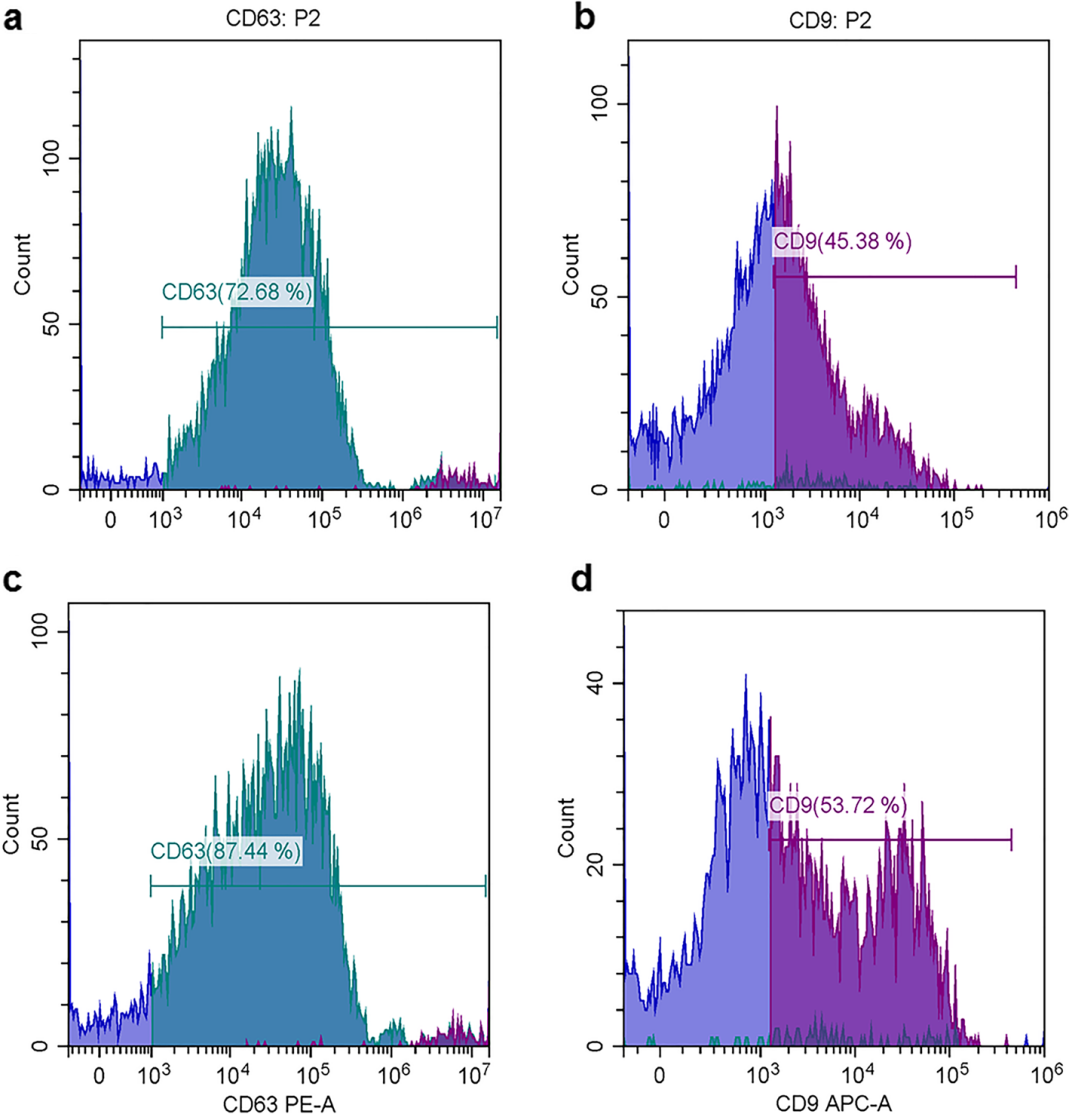
Flow cytometric analysis of tetraspanins (CD63 and CD9) expression in EV subtypes isolated from LVL of healthy (control) and diseased (endometritis) Arabian mares. **A** Histogram representative of CD63 expression in EV subtypes isolated from LVL of healthy (control) mares. **B** Histogram representative of CD9 expression in EV subtypes isolated from LVL of healthy (control) mares. **C** Histogram representative of CD63 expression in EV subtypes isolated from LVL of diseased (endometritis) mares. **D** Histogram representative of CD9 expression in EV subtypes isolated from LVL of diseased (endometritis) mares. Fluorescence (CD63-PE-A and CD9-APC-A, x-axis), vs. number of events (Count, y-axis)

Flow cytometric analysis was restricted to the EVs based on their characteristic properties in the forward scatter (FSC) and violet- side scatter (SSC). Our findings demonstrated that the isolated EVs from serum (Fig. 5A-D) and LVL (Fig. 6A-D) were positive for CD63 and CD9 in both control (Figs. 5A&B and 6A&B, respectively) and diseased mares (Figs. 5C&D and 6 C&D, respectively).

### Increased levels of IL-6, PGF2α and PGE2 in serum in mares suffering from endometritis

**Fig. 7:**
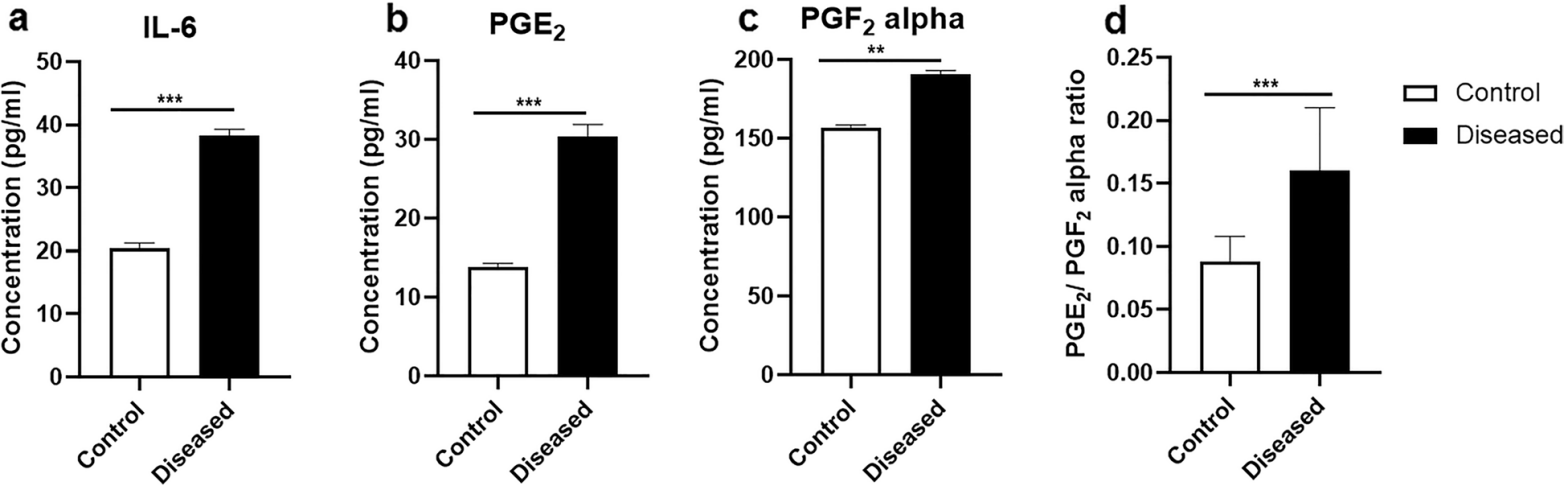
Levels of IL-6 and prostaglandins (PGE_2_& PGF_2α_) in serum of Arabian mares with endometritis compared to healthy (control) mares. **A** Serum concentrations of IL-6. **B** Levels of PGE_2_ in serum. **C** Serum level of PGF_2α_. **D** Ratio of PGE_2_:PGF_2α_ in serum. Data presented as mean ± SEM (*n* = 20/group) Statistical differences among groups are marked with asterisks (^**^P < 0.01, ^***^P < 0.001)

The concentrations of IL-6 (*P* < 0.001), PGF_2α_ (*P* < 0. 01) and PGE_2_ (*P* < 0.001) in serum were higher in mares suffering from endometritis, in comparison with control healthy ones (Fig. 7A-C). Similarly, the PGE_2_:PGF_2α_ ratio was increased (*P* < 0.001) in mares suffered from endometritis, compared to control ones (Fig. 7D).

## Discussion

Extracellular vesicles released from all cell types have temporal and/or spatial effects during different physiological as well as pathological conditions [37]. Therefore, the exchange of EVs cargo between cells favors cell-cell communication and regulation of the immune response [15]. Inflammation of the inner layer of uterine tissues, either due to an infectious agents (bacteria& fungi) or non-infectious agents (trauma& sperm) is the main cause of sub-fertility in mares [24, 38]. The successful resolution of inflammation is a critical step to maintain uterine homeostasis and subsequently provide the optimum milieu for the embryonic development [20]. It is well documented that imbalance between pro-inflammatory and anti-inflammatory mediators is a main reason for impaired uterine function [24, 39]. Until now, the main challenge of equine endometritis is finding a non-invasive, highly sensitive and accurate tool for diagnosis as well as prognosis. So far, endometrial biopsy is the gold standard tool for definitive diagnosis of endometritis in mares, but it is not preferred for most equine owners due to its invasiveness [40].

To the best of our knowledge, this is the first study to successfully isolate EVs from serum as well as LVL of mares suffering from endometrits and compared to control healthy ones. In the current work, the modified serial ultracentrifugation method was suitable for isolation of EVs from LVL, due to the large sample volume [2]. However, the commercial kit was the method of choice for isolation EVs from serum (lower sample volume) [41]. We noticed that sequential increase of the centrifugal force could be the best way for isolation of EVs from LVL, and resulted in heterogeneous EV populations. Our findings were in agreement with previous work by Barranco et al. [2]. Both isolation methods (serial ultracentrifugation and commercial kit) were highly efficient for a good yield of EVs. Furthermore, TEM as well as SEM images showed that the size of EVs typically ranged from 50 nm up to 1000 nm in diameter, and they had classical spherical shapes [3, 11, 42]. The size of EVs was determined by DLS, and this method revealed that diseased mares (endometritis) released more large size EVs, compared to control ones. This notion was supported by previous research, which indicated the disease condition is associated with profound alterations in EVs production and their cargo [43, 44]. Thus, the isolation of EVs and molecular investigation of their cargo may be a promising diagnostic and/or prognostic tool for endometritis in mares. The molecular contents of EVs (proteins and RNAs) that released to body fluids are highly specific and a precious bio-medical tool [45]. Isolated EVs were intact and within the standard range size. Certain surface markers such as anti-CD63 and -CD81 were checked to confirm the purity of isolated EVs. In the current study, the isolated EVs were expressed a high level of CD63 and CD9, which indicates the purification of EVs that were isolated from serum and LVL, as well. Furthermore, ELISA was an appropriate tool for characterization and quantification of isolated EVs from serum and LVL [46, 47]. Additionally, flow cytometry findings confirmed that the isolated EVs from serum and LVL were heterogeneous in size and positive for tetrespanin surface markers (CD9 and CD63). Interestingly, the EVs isolated from serum and LVL in mares with endometritis differentially expressed CD9 and CD63, compared to control healthy ones. This might indicate the presence of distinct EVs subpopulations with significant functional differences in response to uterine inflammation [48, 49]. Also, it was shown that the function of EVs relies on their ability to bind with target cells through certain surface receptors, which are found on each EV subtype [2].

Herein, the serum levels of IL-6, PGF2α, PGE2, as well as PGE2:PGF2α ratio were higher in mares with endometritis compared with healthy ones. This considerable increase in the concentration of these inflammatory mediators could be an indicator of uterine response against inflammation in Arabian mares. Our data are in agreement with previous findings in cows [50], ewes [51], and mares [28, 29, 32]. It is well documented that ER/Golgi route is the classical pathway for secretion of cytokines/chemokines [33, 52]. Additionally, cytokines might influence the biogenesis of EV as well as their content (cargo) [33].

## Conclusion

To the best of our knowledge, this is the first study to successfully isolate EVs from serum and LVL in Arabian mares suffering from endometritis and compared to control healthy ones. Serial ultracentrifugation of LVL and the commercial kit for serum samples were good strategies for isolating a heterogeneous population of EVs with good yield and purity. The diseased mares (endometritis) released larger size EVs compared to controls. Moreover, the EVs isolated from serum and LVL in mares suffering from endometritis were differentially expressed CD9 and CD63, compared to control healthy. Thus, EVs might be used as potential novel non-invasive biomarker for a better understanding the underlying mechanisms associated with developing endometritis in mares. Further studies are required to gain deep insights into the role of EVs during endometritis in equine species.

## Methods

### Chemicals

All chemicals and reagents were obtained from Qiagen (Hilden, Germany), Thermo Fisher Scientific (Wilmington, USA), unless otherwise stated.

### Animals

The study was conducted on 50 Arabian mares (4–8 years old). Mares were divided into; 30 sub-fertile mares suffering from endometritis (diseased group) and 20 fertile mares not suffering from endometritis (control group), (December 2018 to December 2020) at three stud farms near Giza, Egypt. The selected mares showed normal physical parameters and their vital signs (body temperatures, heart rate, and respiratory rate) were within the normal range. Moreover, an orthopedic examination was also done to exclude mares with lameness or active laminitis. None of the mares had dystocia, retained fetal membranes or problems during puerperium. Additionally, none of the mares was in foal heat. All mares were submitted to transrectal ultrasonographic (US) uterine examination using a real-time B-mode machine (Esaote Mylab30- Netherlands) equipped with 5–7.5 MHz linear-array transducer.

It is very important to combine clinical findings and laboratory data when evaluating mares for endometritis [40]. Here in the current work, the criteria for mares to be enrolled in the diseased group (endometritis) were that they had been bred three or more times unsuccessfully in the breeding season, or had a history more than one year of reproductive failure. In addition, the following criteria on a checklist were present: US scanning showed abnormal fluid in the uterus (echogenic or ≥ 2 cm in diameter), positive endometrial cytology, and pathogenic bacterial and/or fungal growth, as well as abnormal gross character of LVL fluid, shown before [40, 53]. Healthy mares (control) exhibited normal breeding history, normal uterine US appearance, and did not show any pathogenic microbial growth for the uterine samples, as well as negative cytology data, and normal gross character of LVL fluid.

### Sample collection

In a sequential manner, samples (blood, uterine swabs and LVL) were collected only once from each mare (1–3 days before insemination) during estrus, after obtaining owner’s permission. Blood samples were collected from the jugular vein. Serum was divided into two portions; the 1st part was kept at − 20 °C for determination of interleukin 6 (IL-6), and prostaglandins (PGF_2α_ and PGE_2_) levels, and the 2nd part was kept at − 80 °C for EV isolation. To prevent degradation of prostaglandins; serum was added to a 1% stabilizing solution of 0.3 M ethylenediaminetetraacetic acid (EDTA) (Sigma) and 1% aspirin (Sigma) [54].

Endometrial swabs were done according to Bohn et al. [55] and Amorim et al. [40] using a sterile double-guarded culture uterine swab (IMV technologies, Paris). After retraction of the swab, one side was rolled onto a clean microscope glass slide for cytological examination, and immediately inserted into sterile screw capped tubes containing 3 ml of Cary-Blair transport medium (Oxoid, USA) for microbiological examination according to Nielsen [56], data not shown.

For cytological examination, slides were fixed and stained with a special commercial cytological stain, Papanicolaou method (Biodiagnostic, Egypt) according to the recommended instruction. Samples were evaluated for cellularity and number of inflammatory cells per 400× field (Zeiss Axioskop microscobe, Carl Zeiss, Thornwood, NY), as well as for any other remarkable features, as indicated by Bohn et al. [55]. Uterine samples were considered as marked for inflammation (endometritis), when the amount of polymorphonuclear leukocytes (PMNs) was greater than 2% as described by Aguilar et al. [57], data not shown.

The LVL was obtained as previously showed by Bohn et al. [55] and Amorim et al. [40], through infusion of around 200 ml of normal physiological saline (0.9% NaCl) into the uterus via equine catheter (IMV technologies, Paris). Afterwards, the original fluid bag that contained LVL (100–120 ml) was kept on ice, till transportation to Lab for further process. The gross character of the LVL fluid: normal (clear) or abnormal (cloudy, discolored, debris) was recorded.

### Isolation and characterization of EVs

Both microvesicles and exosomes were isolated from LVL through serial centrifugation (Supplementary Fig. 3), according to Thery et al. [58], and Gurunathan et al. [46], with some modifications. In brief, LVL from each mare was centrifuged at 2000 xg (Sigma 3-18KS, UK) for 20 min at 4 °C, in order to remove blood and cellular debris. Supernatant then was transferred into new tubes, and centrifuged at 25,000 xg (Thermo Scientific™ Sorvall™ MTX 150 micro-ultracentrifuge, USA) using a fixed angle rotor for 30 min at 4 °C, to get a microvesicles pellet. The microvesicles pellet was suspended in phosphate buffered saline (1x PBS), and then kept at − 80 °C. Afterwards, supernatant was ultracentrifuged at 120,000 xg (Thermo Scientific™ Sorvall™ MTX 150 micro-ultracentrifuge, USA), using a fixed angle rotor, for 190 min at 4 °C to get an exosomes pellet. Then the pellet was suspended in 1x PBS and kept at − 80 °C, for further analysis.

Also, microvesicles and exosomes were isolated from serum using an ExoQuick® Ultra EV kit (System Biosciences, USA). Serum from each mare was thawed on ice, and the extraction procedure was performed according to the manufacturer’s protocol. Afterwards, the isolated EVs were kept at − 80 °C, until further analysis.

### Characterization of EVs

#### Transmission electron microscopy (TEM)

Examination of EVs using TEM was done according to Thery et al. [58] with some modifications. In brief, around 5 μl of sample (three independent replicates/isolation method) were placed onto sheet of parafilm, and then placed onto a carbon-coated 400-mesh copper grid directly from the sample. The grid was placed on a drop of filtered 2% phosphotungstic acid (PTA), and stained for 30–60 s. The grids were dried and the images were acquired using a high resolution electron microscope (HRTEM, JEOL-JEM2100, Japan).

#### Scanning electron microscopy (SEM)

According to Wu et al. [59], EVs samples (three independent replicates/isolation methods) were mounted on a SEM stage by carbon paste, and then a coating of 2–5 nm gold-palladium alloy was applied by sputtering before imaging by scanning electron microscopy (JEOL JSM 5200 SEM Scanning), under beam energies 25 kV.

#### Dynamic light scattering (DLS)

The measurements and particle size distribution of EVs isolated from serum and LVL of healthy (control) and diseased mares were investigated using DLS. Separate pool of serum EVs as well as LVL EV samples (five independent replicates/isolation methods) were suspended in 5 ml 1x PBS, and then the analysis was conducted with a NICOMP 380 ZLS instrument (PSS, Santa Barbara, CA, USA), using the 632 nm line of a HeNe laser as the incident light with a 90^°^ angle and Zeta potential with external 18.9^°^ angle.

#### Detection of surface markers CD81 and CD63

The typical surface markers of EVs: CD81 and CD63 were determined respectively using an ExoELISA-Ultra CD81 (System Biosciences, USA) and ExoELISA-Ultra CD63 kits (System Biosciences, USA), according to the manufacturer’s instructions.

#### Determination of tetrespanins (CD63 and CD9) by flow cytometry

The isolated EVs (three independent replicates/isolation method) were incubated separately with 10 μl anti-CD63 (MX-49.129.5) PE (Santa Cruz Biotechnology, INC, USA), and 10 μl anti-CD9 (C-4) FITC (Santa Cruz Biotechnology, INC, USA) for 30 min at 4 °C. Images were acquired with a CytoFLEX Flow Cytometer, and data were analyzed using a CytExpert Software (Beckman Coulter Life Sciences, USA).

### Evaluation of IL-6, PGF_2α_ and PGE_2_ levels in serum

Serum concentrations of IL-6 were determined by a horse IL-6 ELISA kit (SunLong Biotech Co., LTD, Zhejiang, China), according to the manufacturer’s instructions. The assay sensitivity and range were 0.5 pg/ml and 1.6 pg/ml to 100 pg/ml, respectively. For PGF_2α_ measurement, the commercial PGF_2α_ high sensitivity horse prostaglandin F_2_ alpha ELISA kit (SunLong Biotech Co., LTD, Zhejiang, China) was used, according to the manufacturer’s instructions. The assay sensitivity and range were 0.5 pg/ml and 3 pg/ml to 210 pg/ml. For PGE_2_, the commercial PGE_2_ high sensitivity horse prostaglandin E_2_ ELISA kit (SunLong Biotech Co., LTD, Zhejiang, China) was used in accordance to the manufacturer’s instructions. The assay sensitivity and range were 0.1 pg/ml and 0.8 pg/ml to 50 pg/ml, respectively. The inter- and intra-assay coefficients of variation were 8.6 and 5.5% for IL-6, 6.6 and 10.79% for PGF_2α_, and 6.9 and 12.2% for PGE_2_.

### Statistical analysis

Data are expressed as mean ± standard error of the mean (S.E.M). The normal distribution was checked via Shapiro–Wilk test, Gaussian distribution, Anderson-Darling test, and Kolmogorov-Smirnov test; all data passed the normality test. The Unpaired t test with Welch’s correction (GraphPad Software, Inc., San Diego, CA, USA) was used for statistical analysis of diameters of EVs, IL-6, PGE_2_, PGF_2α_, and PGE_2_/PGF_2α_ ratio between control healthy and diseased (endometritis) groups. *P* values < 0.05 were considered statistically significant. GraphPad Prism 9.0 was used for performing statistical analysis and generating bar graphs.

## Supplementary Information

**Additional file 1 **: **Supplementary Figure 1.** The typical EVs surface marker CD63 was measured using ExoELISA‐Ultra CD63 kit. **Supplementary Figure 2.** The typical EVs surface marker CD81 was measured using ExoELISA-Ultra CD81 kit. **Supplementary Figure 3.** Schematic representation of the protocol used for Evs isolation from LVL in Arabian mares (control& endometritis).

## Data Availability

The data that support the findings of this study are available from the corresponding author upon reasonable request.

## Abbreviations

EVs: Extracelluar vesicles
LVL: Low volume uterine lavage
IL-6: Interleukin 6
PGF_2α_ and PGE_2_: Prostaglandins
TEM: Transmission electron microscopy
SEM: Scanning electron microscopy
DLS: Dynamic Light Scattering
