# Supplementary material for: Extracellular vesicles in low volume uterine lavage and serum: novel and promising biomarker for endometritis in Arabian mares
Source: BMC Vet Res. 2022 Jan 18;18:42. doi: 10.1186/s12917-022-03137-3 (PMC8764842; doi:10.1186/s12917-022-03137-3)
Supplement: Supplementary file 1 — Additional file 1 : Supplementary Figure 1. The typical EVs surface marker CD63 was measured using ExoELISA‐Ultra CD63 kit. Supplementary Figure 2. The typical EVs surface marker CD81 was measured using ExoELISA-Ultra CD81 kit. Supplementary Figure 3. Schematic representation of the protocol used for Evs isolation from LVL in Arabian mares (control& endometritis). [file 12917_2022_3137_MOESM1_ESM.docx]

**Supplementary Materials**

**Supplementary Figures:**

**
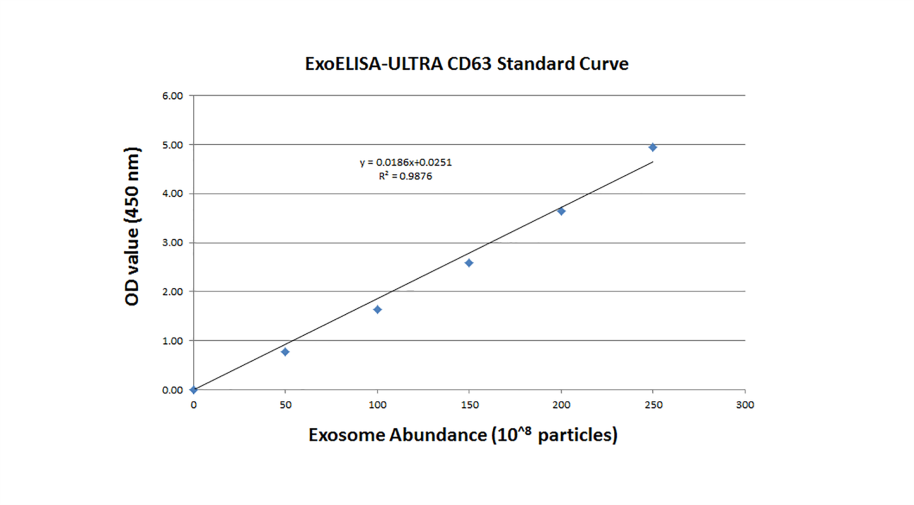
**

**Supplementary Fig. 1:** The typical EVs surface marker CD63 was measured using ExoELISA‐Ultra CD63 kit**.**

**
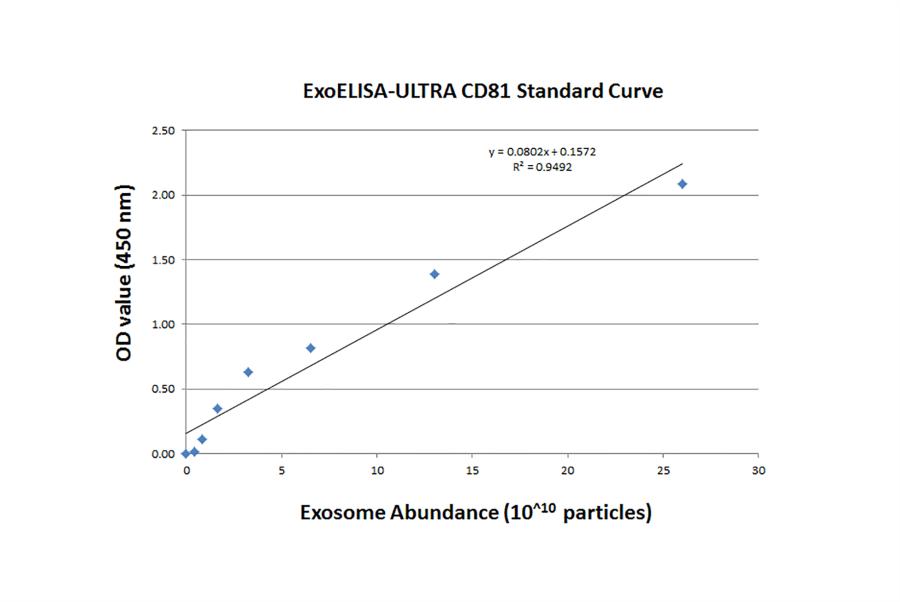
**

**Supplementary Fig. 2:** The typical EVs surface marker CD81 was measured using ExoELISA-Ultra CD81 kit.

**
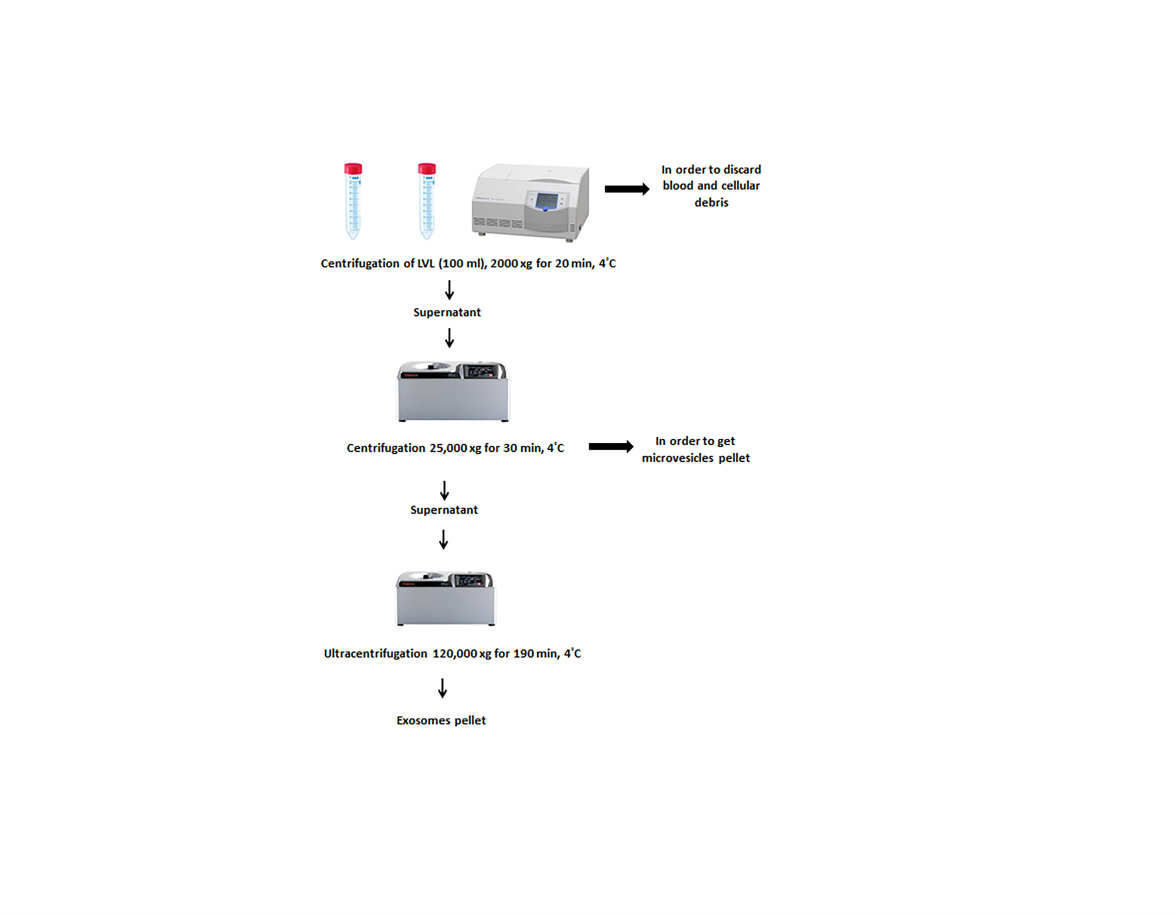
**

**Supplementary Fig. 3:** Schematic representation of the protocol used for Evs isolation from LVL in Arabian mares (control& endometritis).
